# How Numbers, Nature, and Immune Status of Foxp3^+^ Regulatory T-Cells Shape the Early Immunological Events in Tumor Development

**DOI:** 10.3389/fimmu.2013.00292

**Published:** 2013-09-26

**Authors:** Guillaume Darrasse-Jèze, Katrina Podsypanina

**Affiliations:** ^1^Faculté de Médecine, Sorbonne Paris Cité, Université Paris Descartes, Paris, France; ^2^Unité 1013, Institut National de la Santé et de le Recherche Médicale, Hôpital Necker, Paris, France; ^3^Immunoregulation and Immunopathology Team, INEM, Paris, France; ^4^Breast Cancer Biology Research Unit, Institut de Recherches Cliniques de Montreal (IRCM), McGill University, Université de Montreal, Montreal, QC, Canada

**Keywords:** Treg, Foxp3, memory, cancer, tolerance, tumor cells, vaccination, early immune response

## Abstract

The influence of CD4^+^CD25^+^Foxp3^+^ regulatory T-cells (Tregs) on cancer progression has been demonstrated in a large number of preclinical models and confirmed in several types of malignancies. Neoplastic processes trigger an increase of Treg numbers in draining lymph nodes, spleen, blood, and tumors, leading to the suppression of anti-tumor responses. Treg-depletion before or early in tumor development may lead to complete tumor eradication and extends survival of mice and humans. However this strategy is ineffective in established tumors, highlighting the critical role of the early Treg-tumor encounters. In this review, after discussing old and new concepts of immunological tumor tolerance, we focus on the nature (thymus-derived vs. peripherally derived) and status (naïve or activated/memory) of the regulatory T-cells at tumor emergence. The recent discoveries in this field suggest that the activation status of Tregs and effector T-cells (Teffs) at the first encounter with the tumor are essential to shape the fate and speed of the immune response across a variety of tumor models. The relative timing of activation/recruitment of anti-tumor cells vs. tolerogenic cells at tumor emergence appears to be crucial in the identification of tumor cells as friend or foe, which has broad implications for the design of cancer immunotherapies.

## Tumor Recognition by the Immune System: Ignorance, Surveillance, and Tolerance

A now receding branch of tumor immunology literature favors the view that antigens expressed by many tumors would be ignored by the immune system due to inadequate antigen presentation (1–3). Hewitt, after examining the immunogenicity of many tumor cell lines, concluded that only virus-induced tumors are likely to induce an immune response against them (4). Beyond this founding observation, and the reports that many tumor cells do not express MHC proteins, several groups have found functional alterations of the proteasome (5, 6) and TAP (7) in tumor cells and APCs (8), reducing tumor visibility to the immune system. In contrast, many studies have proven that the immune system is activated in the presence of spontaneous tumors (9–12), revitalizing the concept of immunosurveillance.

This concept, first proposed in 1909 (13), was formally defined in 1957 when – based on the findings that the immune system can specifically recognize and reject tumor cells in a chemically induced murine sarcoma model (14) – Burnet proposed that the immune system may prevent tumor development by recognizing antigens absent in normal tissues (15). According to his theory, the immune reactions against tumor antigens expressed by neoplastic cells generally eliminate them at an early stage before any clinical hint of their existence, and frank tumors can grow only after escaping the immune system by diminishing their immunogenicity. The existence of tumor-specific antigens was indeed confirmed in the 1960s by Klein (16). Later, tumors developing in immunodeficient mice were proven to be more immunogenic than tumors developing in immunoproficient mice (17), suggesting that tumors undergo selection by the immune system.

However, in other mouse models, immunodeficiency did not promote tumor development (18–20), and the ability of tumor cells to diminish the expression of their most immunogenic epitopes by adaptation or selection, also called cancer immunoediting, has been questioned (21). The involvement of innate immunity in tumor surveillance was further explored in studies on natural killer cells (NKs), and has produced similar arguments pro (22–24) and con (20, 25, 26). The conflicting accounts on the role of ongoing anti-tumor surveillance produced in animal models are mirrored by findings in clinical studies that measured the risk of tumor development in patients with immunodeficiencies. Numerous publications have observed a significant increase of cancer occurrence in immunodeficient patients, but at the same time there is no study reporting an “explosion” of cancer cases in these patients. For example, in a study of 2005 on a very large number of immunosuppressed renal transplantation recipients, Hollenbeak et al. observed that of the 89,786 patients who underwent transplantation, 246 patients developed melanoma, with an age-adjusted incidence rate of 55.9 diagnoses per 100,000 individuals. This represented an increase in age-adjusted, standardized risk that was 3.6 times greater than the general population (27). Thus, while such studies support a real role of immunosuppression in promoting cancer susceptibility, the risk of developing a melanoma in the absence of a functional immune system, if increased and non-negligible, is still only 0.056%. One interpretation is that tumor development in the absence of immune system is still a rare event, another interpretation is that the immunodeficient state in patients mostly increases risk of cancers of viral etiology, and that the impact of immunosurveillance on preventing non-viral human cancer may actually be relatively minor (28–31). On the other hand, in already established tumors, presence of tumor-infiltrating lymphocytes is correlated with improved survival (32), and intra-tumoral CD8 T-cells infiltration is associated with delayed recurrence and extended survival in oncologic patients (33). A consensus is that the immune surveillance may guard against cancer under certain conditions, but the precise nature of these conditions is unclear.

The first clue that immune tolerance might be a part of the equation came from the works of Nishizuka and Sakakura. While investigating the role of the thymus in tumor immunity in mice susceptible to mouse mammary tumor virus (MMTV)-induced cancer, they observed that neonatal thymectomy at 3 days of age (day 3 nTx) resulted in reduced frequency of breast cancer in tumor-prone (C3H/HeMs × 129/J)F1 females (34), suggesting that cells produced by the thymus after day 3 may protect the tumor. In the following studies, they also looked at tumor development in extra-mammary tissues. There was no increase in the lung and liver tumors after neonatal thymectomy, but the authors reported increased ovarian, lymphoreticular, and pituitary tumor development (35). A notable point of these studies was the discovery that mammary gland development in day 3 nTx female mice was delayed (34) and that mice became infertile secondary to the development of oophoritis (36). At the time, Sakakura and Nishizuka attributed these features to an endocrine role of the thymus, although it is now known to be the manifestation of T-cell-mediated autoimmunity, which paved the road to the discovery of thymic-derived suppressor T-cells, and active tolerance to the tumors.

## Treg-Mediated Tumor Surveillance: Expand to Reign

T-cells capable of suppressing the rejection of implanted tumors were first observed in the late 1970s (37–40). These reports remained underappreciated as were most findings pointing to the existence of suppressor cells, caused, in part, by lack of suppressor-specific cellular markers. The doubts have disappeared only in the 1990s, when Sakaguchi, a former student of Nishizuka demonstrated that CD4^+^CD25^+^ T-cells, baptized “regulatory,” were responsible for the induction of dominant immune tolerance to tumors. First, the transplantable tumors grew in immunodeficient hosts transferred with whole splenocytes, but were rejected in hosts that have received splenocytes depleted of CD25^+^ cells (41). Second, the tumors were rejected following preventive treatments with anti-CD25 antibody (42). In both cases, the presence of CD25^+^ cells inhibited the anti-tumor immune response and their removal led to the complete elimination of the tumor.

In a short time, an impressive number of reports confirmed the association between malignant tumors and the regulatory T-cells (Tregs). Clinical studies have shown that CD4^+^CD25^+^ cells are often present within the tumor mass, and have reported a link between a presence of a tumor and an increase in the proportion and/or the number of CD4^+^CD25^+^ Tregs in the blood (43–47). However some results were more heterogeneous depending of the cancer type, and in some studies, no Treg increase was observed (48). Moreover, sometimes the observed proportion of Tregs seems falsely increased by the reduction of the absolute number of CD4^+^CD25^−^ effector cells (Teffs) (49). Regardless of its causes, an important question was whether the observed increase in Tregs is informative for prognosis. Animal models have argued that Tregs have pro-tumorigenic effects (see above), and tumor volume appears directly correlated to the number of Tregs present in the secondary lymphoid organs in several models (50–52).

Starting with the report correlating presence of Tregs within the tumor infiltrate and a poor survival prognosis in patients with ovarian cancer (Curiel, 200,456), the majority of studies have agreed that an increase in Tregs/Teffs ratio or in an absolute Treg number confers a poor prognosis in cancer patients [see below, and in these recent reviews (48, 53, 54)]. Yet there are instances in which Treg increase is actually linked to a good prognosis, for example in lymphomas (55, 56) and in colorectal cancer (57–59). The reasons for this discrepancy appear to depend on the special nature of these cancers, in which inflammation may promote tumor growth if not regulated by Tregs, but may also be related to a difference in the origin of cells with Treg characteristics observed in individual malignancies.

Concerning the causes of the tumor-induced increase in Tregs, the literature describes several mechanisms: (i) Preferential recruitment of existing thymic-derived Tregs (tTregs), which may be mediated, in part, by chemokines produced by tumors, such as CCL22, that attracts regulatory T-cells, which predominantly express the cognate agonist receptor (60, 61). However, as effector lymphocytes express chemokine receptors as well, chemokine secretion alone cannot explain the preferential recruitment of Tregs to tumor sites (62, 63). The two alternative explanations are (ii) fate conversion – *de novo* induction of peripheral Treg (pTregs) out of effector T-cells; and (iii) clonal expansion – cytokine and/or antigen-induced proliferation in the periphery of tTregs. Given the vast variety of tumor systems in which all these scenarios have been explored, it is conceivable that the nature of the transforming event, or the tissue of origin of the tumor may determine the specific biological mechanism leading to an increase in Tregs.

## Peripherally and Thymic-Derived Tregs in Cancer

Discovered in the early 2000s in mice (64) and in humans (65), pTregs quickly became the subject of active investigation in tumor immunology, generating evidence both for and against their role in tumor tolerance. Adoptive transfer of CD4^+^CD25^−^ T-cells in mice challenged with either colon cancer or B cell lymphoma resulted in induction of CD25 expression in a significant proportion of donor Teffs, as well as appearance of Foxp3 transcript (66, 67).

A major line of research pursued a possible instructive role of TGF-ß, a signaling molecule with pleiotropic functions in both immunity and cancer, and in the conversion of CD4^+^CD25^−^ T-cells to pTreg cells (65). TGF-ß acts by binding to the type II TGF-ß receptor (TGF-ßRII), which is constitutively active as a serine/threonine kinase (68, 69). A CD4^+^ cell-restricted blockade of TGF-ß signaling in mice expressing a dominant negative version of the receptor resulted in eradication of TGF-ß expressing lymphoma or metastatic B16F10 melanoma (70) and has established a firm link between TGF-ß and tumor immune tolerance. In part, such a blockade may impair the pro-tumorigenic conversion to pTregs. Indeed, an *in vitro* study has implied that TGF-ß expressing kidney or prostate tumor cells can stimulate the pro-tumorigenic conversion to pTregs (71). Accordingly, the anti-TGF-ß treatment of mice injected with these tumor cells resulted in fewer tumor nodules; but the *in vivo* experiments did not exclude a possibility of a direct effect of TGF-ß-blockade on RENCA and TRAMP-C2 cell growth. Moreover, pancreatic tumor-derived TGF-ß was shown to activate Foxp3 expression in tumor cells themselves (72). The functional significance of this upregulation is unclear, as in the tumor cells the Foxp3 transcription factor remains restricted to the cytoplasm, contrary to nuclear localization in Tregs, but it may result in a lower immunogenicity of the tumor, as siRNA-mediated inhibition of Foxp3 expression in tumor cells may shift their cytokine expression pattern toward IL-6 and IL-9 secretion (72).

The effect of TGF-ß on the conversion *in vivo* in tumor-bearing mice was addressed more recently using adoptive transfer of CD4^+^25^−^Foxp3^−^T-cells into *Rag*^−^*^/^*^−^ mice. In the presence of a TGF-ß-producing pancreatic Pan02 tumor, the transferred T-cells converted into Foxp3^+^ pTregs, but few FoxP3^+^-converted cells were found when mice were transplanted with a TGF-ß-negative esophageal Eso2 tumors (73). As predicted, the induction of cells with Tregs characteristics in Pan02-bearing mice was blocked by systemic injection of an anti-TGF-ß-antibody. This finding mirrors the clinical situation, when increase of Foxp3^+^ Tregs is observed in patients with a TGF-ß-producing pancreatic adenocarcinoma but not in those with a TGF-ß-negative esophageal tumor (74). Similarly, in non-small cell lung cancer patients, TGF-ß plasma concentrations directly correlated with the frequency of circulating Tregs (75).

As stated above, the spectrum of biological effects of TGF-ß is wide, and is spread beyond the pTreg induction to regulate other Teff responses. For example, anti-TGF-ß treatment significantly and synergistically improved vaccine efficacy as measured by a reduction in growth of the TC1 lung tumor allografts, but anti-TGF-ß alone without vaccine had no impact (76). Moreover, anti-TGF-ß treatment did not affect Treg numbers in lymph nodes and tumors, or their function (76). The resultant synergistic protection induced by anti-TGF-ß plus vaccine combined treatment was likely mediated by CD8^+^ T-cells since anti-CD8 treatment completely abrogated this effect (76). These results, of course, do not exclude a role for peripherally derived-CD4^+^ pTregs, but greatly diminish the chances that CD4^+^ pTregs are the sole culprit behind the TGF-ß effects on tumor tolerance.

Overall, the role of TGF-ß in Treg maintenance is mixed, as it inhibits Teffs and Treg cell proliferation, but is important for tTreg and pTreg survival in the periphery (77). In fact, the nature of TGF-ß/Treg interactions may be more complex than a direct conversion scenario would suggest. For example, a mammary tumor cell line, 4T1, can induce recruitment of TGF-ß-producing Gr-1^+^CD11b^+^ monocytes (78), and a mouse melanoma and a rat colon tumor were shown to convert dendritic cells (DCs) into the TGF-ß-producing cells, which then led to Treg proliferation (79), possibly through a GILZ-dependent mechanism (80). A similar hierarchy of APC/Treg exchange has been clearly demonstrated in colitis. There, DC-produced TGF-ß was shown to be critical to avoid colitis due to its Treg inducing power (81). In this paper, the Sheppard team showed that DCs lacking the TGF-β-activating integrin αvβ8 failed to induce Tregs *in vitro*, and that mice with conditional deletion of αvβ8 in DCs presented reduced proportions of Treg cells in colonic tissue. If It should not be excluded that effector cell expansion may contributes to this observed reduction in the fractional number of Treg cells in the colon, these *in vitro* and *in vivo* results reinforce observations that DCs are essential in the maintenance of both pTreg and tTreg cells in the periphery (82–85).

A major complication that weakens the accounts of *de novo* pTreg induction after adoptive transfer of Teffs in tumor-bearing mice is that the CD4^+^CD25^−^ Teffs subset purified in the majority of the conversion experiments of the pioneer articles, contains around 2% of CD4^+^CD25^−^Foxp3^+^ T-cells that exhibit suppressive functions (86) and can gain CD25 expression and expand after stimulation (87, 88). The experimental approaches based on the sorting of CD25^−^T-cells do not provide supportive evidence for a *de novo* induction of pTregs, and do not exclude a possibility of tumor-driven activation and expansion of CD4^+^CD25^−^Foxp3^+^ thymus-derived tTregs. Accordingly, experiments using Teffs transfer from donor mice expressing a Foxp3-reporter indicate that generation of peripherally derived FoxP3^+^ pTregs out of GFP^−^ Teffs within tumors is inefficient and that tumor-infiltrating GFP^+^FoxP3^+^ tTregs are highly stable and do not readily convert back to FoxP3^−^ T-cells contrary to pTregs (89). Some authors suggest that proliferating Helios^+^ Treg cells are a major population in tumors (90), which may be interpreted against pTregs conversion in tumors, Helios being a tTreg marker (91, 92). But Helios may be upregulated in peripherally derived pTregs after activation by DCs (93).

Another line of evidence questioning the primary role of pTregs in tumor tolerance comes from a recent paper describing the tolerogenic response against the prostate-associated MJ23 self-antigen expressed by prostate tumors induced by an SV40 TAg transgene. On an immunoproficient background, these tumors are infiltrated with MJ23 tumor-specific Tregs, but no MJ23 tumor-specific Tregs were found in tumors that have developed in *Aire*^−^*^/^*^−^mice. As Aire is important for tTreg development but dispensable for pTreg induction, these findings indicate that the tumor-infiltrating Treg cells specific for the highly immunogenic MJ23 are principally of the thymic origin (94).

Overall, there is little doubt that pTregs may appear from CD25^−^ subsets, probably from recent tumor emigrant cells (95), in the presence of tumors under certain experimental conditions. Whether this subset plays a substantial role during spontaneous tumor development, is less clear. The difficulty is best illustrated by recalling the original report of Sakaguchi, showing that the immunodeficient mice reconstituted with CD25 depleted splenocytes acquired efficient anti-tumor responses in various cancer models (41). Stated otherwise, any spontaneous pTreg conversion that may occur in this experimental setup does not prevent clearance of the transplantable tumors.

## Activated/Memory Tregs in the Early Immune Response to Cancer

When we were studying the kinetic of early immune responses in various models of cancer by adoptive transfer of CFSE-labeled T-cells, we were struck by the fact that Treg response was not a late event, secondary to the activation of IL-2-releasing anti-TAA effector T-cells (Tumor-associated antigen-specific Teffs), but was actually a very early event, preceding any Teffs activation (52). Such a rapid tumor-specific response of the immune system was counterintuitive in a model of primary tumor exposure, but it bore well with the earlier reports that tumor growth can activate immune cells very quickly.

**Figure 1:**
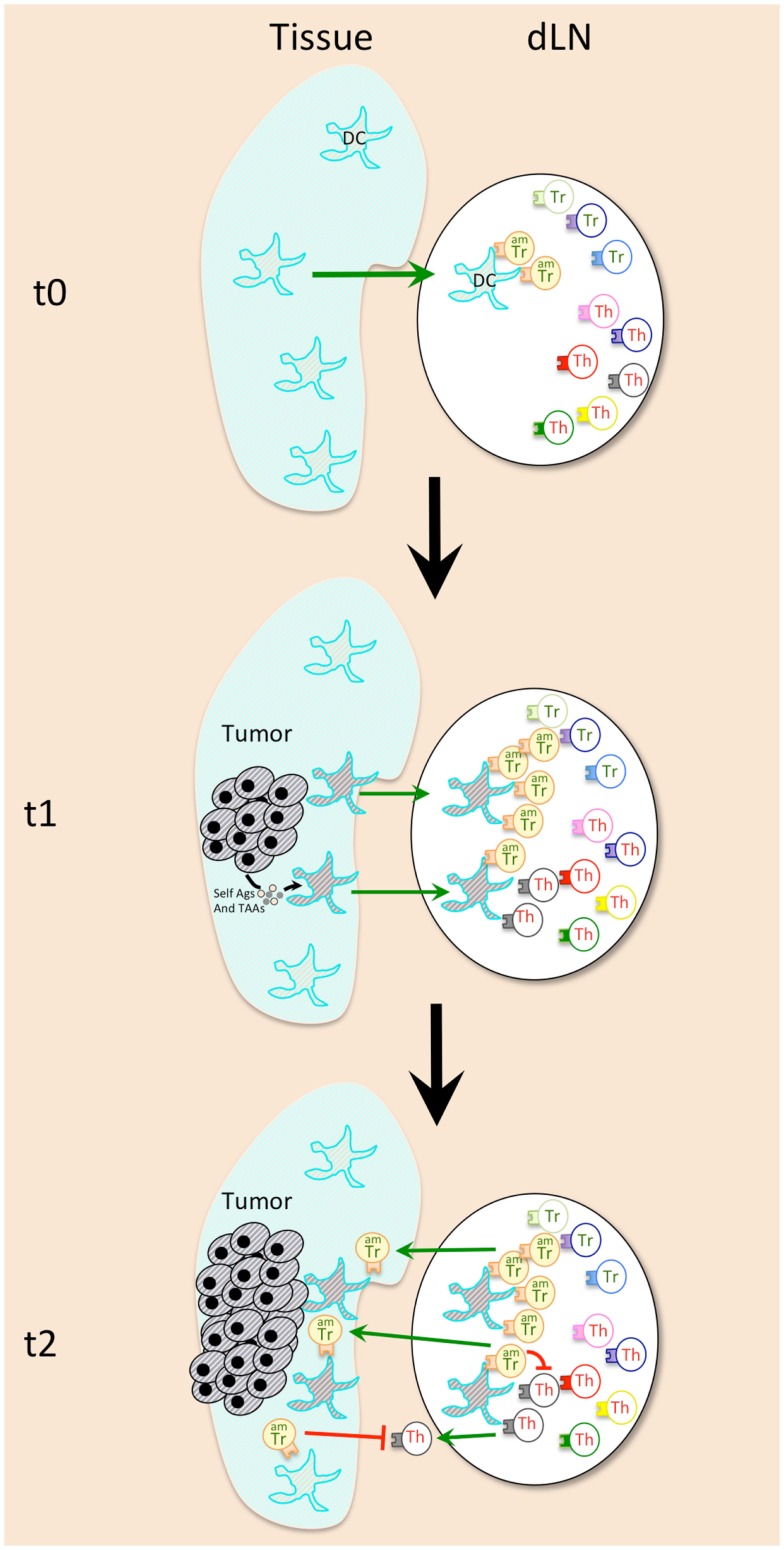
**Early events during cancer emergence lead to immune tolerance against tumor**. Activated memory Tregs (AmTregs or amTr, beige lymphocytes) are the first to be stimulated by the presence of the tumor (gray round-shaped cells) via recognition of self-Ag presented by dendritic cells (DCs, star-shaped cells) coming from the tumor site (t1). AmTreg will then proliferate faster than TAA-specific Teffs (Th, gray lymphocytes) that are naïve (or have already been suppressed at the steady state). AmTreg will then inhibit either Teff activation, proliferation, migration, and function either/or DCs presentation and costimulation (t2).

In 1975, Bhatnagar and colleagues have measured *ex vivo* thymidine incorporation by splenocytes and detected substantial cellular immune responses as early as 1–2 days after i.p. injection of methylcholanthrene-induced fibrosarcoma cells (96). The intensity and rapidity of the cellular response was dependent of the number of cells injected and was always followed by a gradual loss of cellular reactivity against the tumor cells. The progressive loss of immune recognition for tumor cells correlated with progression of tumor growth (96). These observations were confirmed by Berendt and North, who provided evidence supporting the hypothesis that immunity to tumors declines with time as a result of T-cell-mediated immunosuppression (40). More recently, in several injected tumor models (B16 melanoma, 4T1 carcinoma, AB1 mesothelioma, and more) and in an inducible-oncogene-driven breast tumor model, an increase in T-cell division was detected as soon as 2 days after the emergence of the tumor by measure of CFSE dilution as well as BrdU incorporation (52). But this early response was restricted to CD4^+^CD25^+^Foxp3^+^ regulatory T-cells, and it appeared to precede the response of conventional T-cells (Figure 1). The responding Treg cells were specific to the antigens, which, although expressed by tumors, were already present in mice before tumor appearance (Figure 1). In other words, the tumor-derived antigens able to stimulate Tregs were self-antigens. Indeed, no Treg expansion was observed against tumors that were not bearing a cognate self-antigen recognized by the transferred tTregs. These observations confirmed previous observations that the self-specific Tregs suppress anti-tumor responses (97, 98), although it did not exclude a possibility that Tregs specific for tumor neoantigens may also participate to the induction of tolerance to the tumor (99, 100). Recently, it was demonstrated that Aire-mediated expression of peripheral tissue antigens drives thymic development of a subset of organ-specific tTregs, which are likely recruited by tumors developing within the associated organ (94).

Concerning the APCs that may be responsible for presentation of the tumor self-Ags to Tregs, the good candidates are tissue DCs, which are known to be especially potent in stimulating and maintaining the actively dividing Treg pool (83). Indeed, DCs from tumor-bearing mice were shown to recruit Tregs and to favor their proliferation in the draining lymph nodes (79) (Figure 1). These DCs may present antigens derived from proteins secreted by the live tumor cells, or those derived from tumor cells that die during transformation-induced apoptosis. Of note, microvesicles that are released by tumors and may be captured by DCs for tumor antigen presentation (101) appear to have a role in Treg expansion and activation (102) (Figure 1). Moreover, Treg subset expands after adoptive transfer in MHCII^+/+^ but not in MHCII^−/−^ tumor-bearing mice, which proves that cytokines released in the tumor-bearing mice are not sufficient by themselves to favor Treg recruitment, and that antigen-driven proliferation is mandatory (83).

Isolation of Tregs with activated/memory vs. naïve phenotype from tumor-free mice followed by adoptive transfer to tumor-bearing mice showed that the initial proliferation of Tregs in tumor-draining lymph nodes was confined to the pool of activated/memory Tregs (amTregs) present in naive mice, (52). These cells were previously characterized as an activated/memory subtype of Tregs, constantly stimulated by self-antigens at the steady state (103). These amTregs are phenotypically and functionally distinct from naïve Tregs (103, 104), and are highly potent at suppressing autoimmune responses (105, 106). The intensity of the early anti-tumor Treg response is thus explained by their self-specificity and activated/memory status.

The early dividing cells described in tumor-bearing mice since 1975 are thus the tolerogenic amTregs cells, a conclusion that is further confirmed by observing tumor rejection following short-term depletion of proliferating immune cells via early administration of anti-mitotic hydroxyurea (HU) or cyclophosphamide (CY) in mice bearing HU/Cy-resistant tumors (50, 52, 107, 108). The early administration of these drugs has a much stronger effect than the late administration, once again suggesting that the immune cells that divide early in the presence of an emerging tumor favor tolerance. Accordingly, a recent analysis of Treg subsets in Her2/Neu-expressing mammary tumor-bearing mice revealed the existence of a Cy-sensitive CD4^+^Foxp3^+^CD25^+^ subset with tumor-seeking migratory phenotype, characteristic of amTregs, and capable of high avidity T-cell suppression (109). In addition, the tumor-infiltrating Foxp3^+^ T-cells express high levels of memory/tumor-associated CCR8 and CXCR4 receptors, and antigen priming is required for the induction of this trafficking receptor phenotype. Thus only antigen-primed, but not antigen-inexperienced naive, FoxP3^+^ T-cells can efficiently migrate into tumors (89). Of course, the effector T-cells also start to proliferate after an adoptive transfer into tumor-bearing mice, but with a primary kinetics that is much slower (9–12 days) than that observed in Treg subset (2–4 days) (83). This delay appears to be sufficient for the establishment of a stable immunosuppressive environment.

To test if tolerance to tumors was due to the Treg/Teffs imbalance induced by the delays between their respective activation/expansion, we adoptively transferred high numbers of HA-specific Teffs in mice bearing HA-expressing tumor cells. We observed complete remission in mice adoptively transferred with antigen-experienced HA-specific Teffs (52). Complete regression was also found (i) in secondary-challenged mice cured from first tumor challenge by temporary Treg-depletion (42, 52) and (ii) in tumor-pre-immunized mice (52, 110–112). The activated/memory Teffs, are able to eradicate very efficiently even poorly immunogenic tumors like B16 melanoma (110, 111), regardless of the number of Tregs present in the mice (52). Even highly suppressive adoptively transferred tumor-specific Tregs are not able to reverse the anti-tumor memory response (52). The resistance of activated/memory Teffs (amTeffs) to Treg-mediated suppression demonstrated was also observed in other conditions like allograft rejection (113) and autoimmune inflammation (114). Nishikawa and colleagues also observed that CD45RO^+^ but not CD45RA^+^ tumor-specific CD4 T-cells from cancer patients were resistant to Treg suppression (115). This resistance could be due to the fact that activated Tregs can downregulate expression of costimulation molecules by DC (116), but activation/function of amTeffs is much less dependent on costimulation than that of naive T-cells (117). Together, these observations suggest that anti-tumor amTeffs could be inherently more resistant to Tregs, and explain why detection of amTeffs correlates with good prognosis in cancer patients (118, 119).

The memory status of Treg and Teffs in early tolerance induction might be important in other settings than just cancer. Several analogies between pregnancy and cancer [reviewed in (120)] point to similarities between the early Treg responses to embryo implantation and tumor emergence. In a just-released study, we observed that early Treg responses to embryo implantation obey to the same rules as those in cancer setting: Tregs expressing markers of the amTreg subset are rapidly recruited to para-aortic conceptus-draining lymph nodes and are activated in the first days after embryo implantation in both syngeneic and allogeneic matings (121). They are also at least in part self-Ag specific, as seen in tumor emergence. Finally, pre-immunization against paternal tissue Ags results in the increase of aborted fetus frequency, and additional Treg-depletion (by anti-CD25) at the time of pre-immunization against paternal tissue Ags, leads to very high frequencies of fetus loss (121). Thus, thymic-derived amTregs appear as a driving force of tolerance to self-ambiguous tissues in the absence of infectious danger signals or pre-immunization.

One can then wonder how an immune system that protects deadly tumor cells may survive evolution. We speculate that the AmTreg tolerant response has been actually positively selected to protect allogeneic fetuses against immune rejection. Indeed, Foxp3-expressing Treg-like cells appeared in the first live-bearing animals like *Tetraodon* (2400 million years) (122) and zebrafish (123), both histotrophic viviparous species. Tregs were thus probably selected in part to protect allogeneic fetuses against immune rejection (121, 124), but the pro-tumorigenic activity of Tregs was not counter-selected because cancers mostly develop late in life (125) without affecting reproductive life span.

## Implications for the Design of Anti-Cancer Immunotherapies

**Figure 2:**
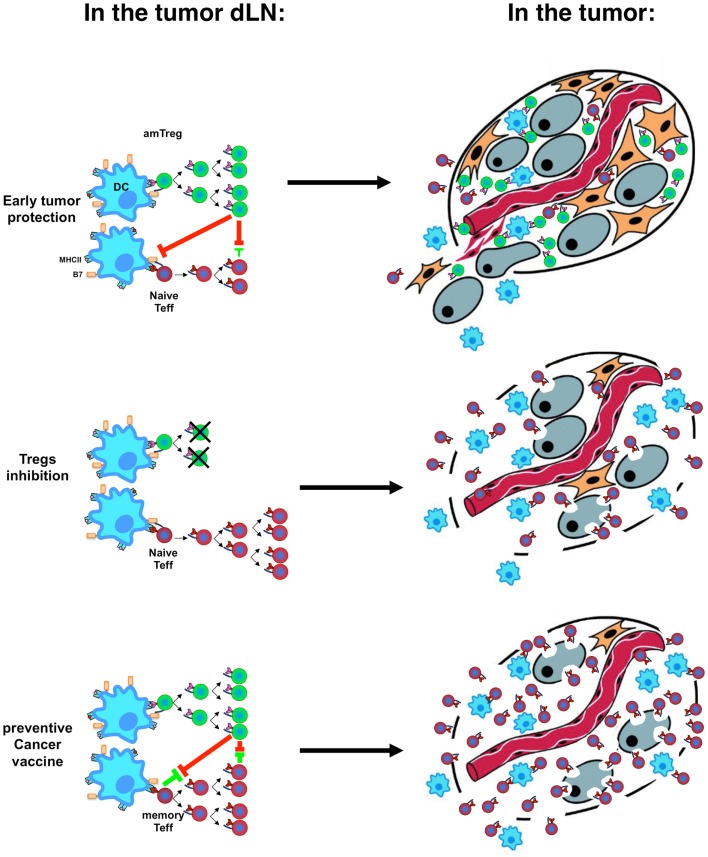
**Immune tolerance vs. immune rejection decision process**. Activation kinetics and memory status of Tregs (green) and Teffs (red) in the tumor-draining lymph nodes (dLNs, left) after stimulation by dendritic cells (DC, blue) result in the infiltration of the tumor by different cell subsets with different speed and different tumor fate (right, with tumor cells in gray).

Activation kinetics and memory status of different T-cell subsets at tumor emergence are pivotal in the outcome of cancer (Figure 2) and explains why preventive immunization is more effective than therapeutic immunization and suggests (i) that preventive vaccination against cancer should be considered seriously and (ii) that therapeutic vaccination could actually worsen host tolerance to tumor antigens (126, 127). Development of vaccination strategies must include treatments aimed at Treg-depletion (128–130) or at inhibition of their function (131–133), with mandatory validation of the effect of therapeutic vaccination on the level/function of Tregs. Preventive vaccination with tumor-specific antigens presented in a context that would not stimulate amTregs will improve development of efficient amTeffs, which may mount efficient effector responses when a tumor emerges.

Noteworthy, although amTeffs are resistant to Tregs, and can cure mice if provided at the time of tumor implantation, the global immunosuppressive environment established by Tregs in draining lymph modes and at the tumor site (134) can develop to a point where later therapeutic administration of amTeffs would no longer be effective (52).

Together with vaccination and beyond, ablation of Tregs in cancer patients appears to be a promising direction, especially if performed early in the course of the disease (129, 135). Nonetheless, we need to remember that the efficiency of anti-tumor responses after Treg ablation is certainly tumor- and genetic background- dependent: Treg ablation results in minimal rejection and delayed growth of B16 tumors in B6 mice, 60% rejection of 4T1 tumors in BALB/c mice (83), and close to a 100% rejection of RL♂1, MOPC-70A, and Meth A tumors in BALB/c mice (41, 42). These diverse outcomes may depend upon (a) the percentage of Treg cells in a given strain of mice in the steady state, (b) the natural ability of some mouse backgrounds to favor strong Th1 responses, and (c) the tumor-specific expression of immunodominant antigens able to trigger strong anti-tumor effector responses (136). These observations from tumor-bearing mice must be kept in mind while designing new immunotherapies strategies in cancer patients.

Altogether, these recent discoveries on the events taking place during the early tumor immune response highlight the importance of the timing and kinetic of Treg and Teff engagement, which depends on their memory status (Figures 1 and 2). In theory, this may disqualify tumor-induced pTregs from playing a substantial role during the early tumor development as they arise preferentially from naïve recent thymic emigrants (95). This does not exclude their eventual involvement in some later events that may sustain the ongoing tolerance. But the fate of the tumor is being decided early, pTregs are unlikely to have much impact in most cancers. Their late arrival in the battle and the absence of memory status puts pTregs at disadvantage during the early tumor development. In tumor immunology and beyond, the timing of engagement dictates the final outcome of an immune response.

## Conflict of Interest Statement

The authors declare that the research was conducted in the absence of any commercial or financial relationships that could be construed as a potential conflict of interest.
